# Metonymic event-based time interval concepts in Mandarin Chinese—Evidence from time interval words

**DOI:** 10.3389/fpsyg.2022.896003

**Published:** 2022-08-02

**Authors:** Lingli Zhong, Zhengguang Liu

**Affiliations:** School of Foreign Languages, Hunan University, Changsha, China

**Keywords:** time conceptualization, event-based, metonymic conceptualization, time interval words, Mandarin Chinese

## Abstract

Starting from the overwhelming view that time is metaphorically conceptualized in terms of space, this study will, on the one hand, take the time interval words into minute analysis to confirm our view of event conceptualization of time at a more basic level along with space–time metaphoric conceptualization of time at a relational level. In alignment with the epistemology of the time–space conflation of the Chinese ancestors, our view is supported by the systematic examination of evidence related to the cultural origins of the conceptualization of time, through a scrutiny of the original meanings and construction of words related to intervals of time in Mandarin Chinese. This study offers a new explanation of how: (1) the conceptualization of time in Chinese is realized through metonymic cognition and (2) words related to specific intervals of time are coined based on the metonymic conceptualization of related events or a corresponding event schema. Five major types of event-based metonymies are identified, and their interactive functions are illustrated. Based on this evidence, we argue that the double nature of both metaphoric and metonymic time conceptualization in Mandarin Chinese lies in the fact that time interval words can be used in its time categorial sense or as a time entity which suggests the etymological origins of Chinese as ideograph. It is concluded therefore that the event-based metonymy conceptualization of time can provide better insights into the characteristics of Chinese modes of thinking and its influences on the perception of and interaction with the world. This study can also serve as good evidence for the shaping effect of language on cognition.

## Introduction

Metaphor and metonymy are two of the four comprising structures of the Idealized Cognitive Models or ICMs (Lakoff, 1987, p. 68). Lakoff defined ICM as a complex structured whole, a gestalt, the way whereby we structure our knowledge of the world, i.e., they are cognitive organization tools that help us represent reality in an idealized way (Lakoff, 1987, 1989). Within Cognitive Linguistics, the distinction between metaphor and metonymy was originally drawn by Lakoff and Johnson (1980: 35–40) and has been further discussed by many linguists. However, Ruiz de Mendoza (1999), Ruiz de Mendoza and Pérez (2001), and Ruiz de Mendoza and Peña (2008) pointed out that, in essence, the sole (and crucial) distinguishing feature between metaphor and metonymy is to be found in the domain-internal nature of metonymic mappings which contrasts with the domain-external nature of metaphoric mappings. In other words, metaphors are mappings across two conceptual domains (the source and the target), whereas metonymies are built upon a single domain in such a way that one domain is already part of the other *via* expansion (source-in-target metonymy, as in saying “hand” for “laborer”) or reduction (target-in-source metonymy, as in “She tied her shoes” for “She tied her shoelaces”) operations. In this connection, Ruiz de Mendoza and Peña (2008) managed to explain how conceptual metonymic chains (i.e., double vs. triple metonymy, involving a double or triple expansion or reduction process) operate across multiple domains.

Conceptual metaphor and metonymy are among the most important tools in the process of cognitive exploration of the nature of time. As an abstract concept, time must be understood and represented by some familiar, concrete concepts of experience. As Barbara (2016: X) argues, time cannot be directly researched, one has to approach it in terms of categories of the mind which find their manifestation in the linguistic construction on a number of levels. Research has shown that the use of spatial expressions for notions of time has been attested in many of the world’s languages. This ubiquitous phenomenon may in part be motivated by our common experience of space and time (Radden, 2011). As Kronasser (1968: 158) has observed, “in our everyday life, there is no experience of space without time nor an experience of time without space.”

Previous studies about time conceptualization or representation can be roughly categorized into three major areas, that is, the way and the pattern of conceptualization as well as the universality or cultural differences. The dominant view today is that time is conceptualized spatially in a broad range of languages and cultures, and a few basic metaphoric mappings from the spatial domain to the temporal one recur in language after language (Núnez and Sweetser, 2006). But some scholars found there is big cultural differences as to the way and pattern of time conceptualization (Sinha et al., 2011; Silva Sinha et al., 2012; Sinha and Gärdenfors, 2015; Silva Sinha, 2019). Moore (2014a, p. 4) cites Evans (2013a) and Galton (2011) that “time is very different from space, so temporal metaphor does not involve similarity between time and space, or a dependence of temporal concepts on spatial ones, though metaphors of space and motion are indispensable for reflecting on temporal experience.” In English spatial terms like "front and back" are used to talk about time, the terms used to order events are the same as those used to describe asymmetric horizontal spatial relations.

## Time-line: One or two space–time metaphoric systems?

It is widely recognized that English has two space–time metaphoric systems to sequence events in time (McTaggart, 1908; Clark, 1973; Lakoff and Johnson, 1980; Boroditsky, 2001, 2008, 2011b). One is the *ego-moving* metaphor, which means the speaker or observer moves along the timeline toward the future (*We are coming to the end of the year; We are coming up on Christmas*). The other is the *time-moving* metaphor (*The end of the year is coming; The Christmas is coming up*), wherein times as entities moving with respect to a static experiencer or wherein time is conceived of as a river or conveyor belt on which events are moving from the future to the past (*The reception is after the talk*; Gentner et al., 2002). These two metaphors lead to different assignments of front and back to a timeline.

But in Mandarin Chinese, both vertical and horizontal terms are used to talk about time, however, the crucial difference is Mandarin speakers appear more likely to speak and think vertically than English speakers and also rely more heavily on time-moving (as opposed to ego-moving) metaphors than do English speakers (Boroditsky, 2001, 2008, 2011b). The main focus of Boroditsky’s studies is on the time as a relational concept, since her experiments intend to explore the difference in how English and Mandarin Chinese talk about or think about time.

The two space–time metaphor systems lead to the very tricky and controversial issue of the time direction of *qian* (前front) and *hou* (后back) in Chinese as summarized into three views by Yu (2012): (a) the ego faces the past (Huang, 1981; Alverson, 1994); (b) the ego faces the future (Yu, 1998); and (c) the ego faces both the past (the primary, preferred case) and the future (the minor case; Ahrens and Huang, 2002; Zhang, 2003; Zhang and Luo, 2007; Wang, 2016).

## Ideographic time interval words: Both time in its categorial and etymological senses

In actual use or speech context of Mandarin Chinese, when time is intended in its categorial sense, it is spatially conceptualized in terms of the direction of motion in space, like “春已来临 (*Spring has arrived*).” In such cases the timeline for the “Time-moving metaphor” flows as in English like *Christmas is approaching*. The time interval word *chun* (春spring) is used here to talk about its relationship to the other seasons of the year. It’s a relational concept.

Besides, when time interval words as they are in the lexicon can also be regarded as the time entity or time units. Then Chinese speakers can, on the one hand, use them in their categorial sense, and at the same time can also just take them as words and trace their etymological origins and meanings. This will be what the present paper is concerned here, that is, the paper will illustrate the way time interval words etymologically get their meanings from their component parts rather than they are used in a relational sense when we are talking about time.

As Janda (2011) has shown the mapping processes word combinations go through are actually metonymic mappings rather than metaphorical. But the difference between Janda’s and our work here is that Chinese time interval words go through event metonymical mapping rather than entity metonymic mapping.

## Spatial-time conceptualization and eventive-time conceptualization

### “TIME IS SPACE” as a deep metaphor

The conceptualization of time through space has been the basic consensus of past research. The conceptual metaphor “TIME IS SPACE” is considered as a deep metaphor for all human being (Lakoff and Johnson, 1999, p. 139; Fauconnier and Turner, 2008, p. 56). Scholars have provided plentiful evidences from the following three aspects: (1) Evidence from languages and cultural artifacts (Clark, 1973; Traugott, 1978; Tversky et al., 1991; Haspelmath, 1997; Evans, 2003, 2005, 2013a, 2013b; Levinson, 2003; Moore, 2006, 2011, 2014a,b, 2017; Boroditsky and Gaby, 2010; Fuhrman et al., 2011; Radden, 2011; Tenbrink, 2011; Núñez and Cooperrider, 2013; Pamies-Bertrán and Yuan, 2020); (2) Evidence from co-speech gesture or other non-linguistic thought (Casasanto and Lozano, 2006; Núnez and Sweetser, 2006; Cooperrider and Núñez, 2009; Casasanto, 2010, 2016; Fuhrman and Boroditsky, 2010; Casasanto and Jasmin, 2012; Levinson and Majid, 2013; Bender and Beller, 2014; Cooperrider et al., 2014; Walker and Cooperrider, 2016; Li, 2017); (3) Evidence from private mental representations (Boroditsky, 2000; Boroditsky and Ramscar, 2002; Kemmerer, 2005; Núñez et al., 2006; Casasanto and Boroditsky, 2008; Margolies and Elizabeth, 2008; Ding et al., 2020).

### Conceptual metaphor is not the only means for the understanding of time

Although previous study (Buzsáki and Tingley, 2018) has provided neuroscientific evidence and theory for the universalist hypothesis of the cognitive capacity for space–time mapping, some studies have demonstrated that a complex of factors, such as thinking about the experience of spatial motion (Alloway et al., 2001; Boroditsky and Ramscar, 2002), fictive motion (Matlock et al., 2005; Richardson and Matlock, 2007) and cross-cultural contexts, language and personality differences, event valence, writing direction, emotional experiences and lifestyle (Gentner et al., 2002; Margolies and Elizabeth, 2008; Boroditsky, 2011a,b; Richmond et al., 2012; Duffy, 2014; Duffy et al., 2014; Duffy and Evans, 2016; Qin, 2021) may also influence people’s perspectives on the movement of events in time, how they think about and organize time and choose metaphorical systems. For example, Boroditsky and Gaby (2010) found that Pormpuraaw of Australian use absolute spatial reference systems to represent time along an east–west axis rather than using a relative spatial axis provided by their bodies as in other cultures. Fedden and Boroditsky (2012) investigated the representations of time among the Mianmin of Papua New Guinea and found that Mian lacks the standardized time representation as in the western developed industrial society, and it is replaced by evidence for a variety of temporal representations. These results extend the previous work on spatial representations of time to a new geographical region, physical environment, and linguistic and cultural system.

Furthermore, the debate arising from the experimental studies of temporal metaphor seems to be inconclusive: The prevailing view on talking about time, as it seems, is that English speakers often use horizontal spatial metaphors, whereas Mandarin Chinese speakers use both vertical and horizontal spatial metaphors. Boroditsky (2001) showed that while Chinese–English bilinguals speakers were faster to verify a temporal target after they had seen a vertical spatial prime rather than a horizontal one, whereas English monolinguals showed the opposite pattern, thus supporting the linguistic relativity hypothesis. This finding prompted a negative response in the results obtained from six experiments of January and Kako (2007) for a wider range of English monolinguals. Further studies conducted by Tse and Altarriba (2008) supported the initial claim, in part, for the Chinese-English bilinguals, but the similarity in the pattern of findings for both Chinese–English bilinguals and English monolinguals refutes the linguistic relativity hypothesis.

Bernárdez (2013) holds the view that “Conceptual Metaphor is not the only means at our disposal for the understanding of our experience(s) of time, it also has to be remembered that CMT cannot be accepted as “proven” and significant criticism on it is frequently found.” Murphy (1996), Strauss and Quinn (1997) and McGlone (2007) have successively put forward some critical views on CMT from different perspectives, while Ruiz de Mendoza and Pérez (2011) offered a critical review of these usual criticisms. Boroditsky and Ramscar (2002), on the other hand, argue that spatial-related concepts could (but not must) facilitate the understanding of certain temporal concepts. This latter view could be regarded as a weaker version of space–time conceptual metaphors, which is also consistent with the authors’ argument that temporal-related concepts might be represented by other non-spatial means or expressions.

Núnez and Sweetser (2006) have confirmed that Aymara lacks the conceptual metaphor of Ego-RP Moving Time. More recent seminal studies have demonstrated that neither the linguistic space–time metaphor nor the existence of a “mental timeline” manifested in speech, gesture or sign appear to be universal (Sinha et al., 2011; Le Guen and Balam, 2012; Silva Sinha et al., 2012; Sinha and Gärdenfors, 2015; Le Guen, 2017; Silva Sinha, 2019). These findings appear to be fascinating contrast to these well-known patterns and challenges to the cross-cultural universality of metaphorical cognition that have been slowly accumulated in our databases.

### Event-based time: A new perspective for the understanding of time

In the Amazonian cultures and languages, time is conceptualized exclusively in terms of events that have no numerically based metric time systems such as calendar or clock time. Not only was there no evidence of a linguistic MT, but the metaphors for time were based not on spatial direction and orientation, but on embodied perception and cognition (Silva Sinha et al., 2012; Sinha and Bernárdez, 2015; Silva Sinha, 2019).

As for the notion of “Event-based time,” it is claimed that event-based time units are *non-metric* (as in the case of “clock time” and “calendar time,” *cf.* Levine, 1997; Postill, 2002), and are defined by the events from which their names usually derive (Silva Sinha et al., 2012; Sinha and Bernárdez, 2015) and may be based upon natural cycles of events, such as the diurnal and seasonal rhythms or on social norms and conventions (Silva Sinha, 2019). Event-based time interval terms may refer to the interval either as a reference point, or landmark in time, as for example “sowing or harvest time.” The duration of the interval of time referred to the duration of events, as for example the Chinese expressions “一袋烟/ 一盏茶/ 一炷香/ 一顿饭的工夫 (*yi dai yan/ yi zhan cha/ yi zhu xiang/ yi dun fan de gong fu*),” which means “the time it takes to smoke a pipe, drink a cup of tea, burn a wick of incense or have a meal.” There are also expressions such as “弹指*tan zhi* (snap one’s finger),” “刹那*sha na* (instant moment),” which are “short-term quantifiers” that originating in Buddhism, where it is generally believed that there are ‘six snaps of fingers’ in 1 min. (the length of time taken to click the fingers once represents 10 “moments” and six clicks of the fingers represents 1 min.) and 10 instant moments in “a mere snap of the fingers.”

Núnez and Cooperrider (2013), p. 220 believe that “The human story, as it has unfolded over thousands of years in different parts of the world, has largely centered on everyday activities like food gathering and preparation, tool making, shelter construction, and the anticipation and observation of rites and rituals. These and other basic human endeavors depend for their success on a robust understanding of temporal relations.” This story was echoed in ancient China, where the concept of time was understood by the ancient Chinese precisely from their full understanding of the relationship between time and events. According to Wang (1990), p. 6, “In the earliest primitive society, there must have been a stage in which humans had no concept of time. It was only under the repeated ‘enlightenment’ of nature, such as the rising and setting of the sun, the wax and wane of the moon, and the alternation of cold and warmth, that humans finally came to realize the concept of ‘time’.” From the cultural origin of time conceptualization in Mandarin Chinese and the character-making evidence of time-interval words, it can be inferred that the conceptualization of time by the ancient Chinese features two basic approaches. One is that they gradually developed their concept of time from their observations of the universe and daily life, and the other is the iconic evolution of their observations and life experiences through metonymy.

## Materials and methods

The above-mentioned time interval words jointly point to a fact that ancient Chinese conceptualization of time is more metonymic in nature, which serves as the focus of the paper. But due to space limitations, based on systematic examination of evidence related to the cultural origins of the conceptualization of time, this study is targeted at demonstrating how metonymy works in such kind of linguistic evidence for event-based metonymic conceptualization of time in Mandarin Chinese from the original meaning and glyph structure of some of the most commonly used time interval words. These words are chosen from three authoritative Chinese dictionaries, Shuowen Jiezi^*^*^1^, Dictionary of Time-reckoning and *Dictionary of Time Category in Ancient Chinese* (Wang, 1999, 2004), which are regarded as the most authoritative Chinese dictionaries for recording and studying the concept of time in Mandarin Chinese, and these time interval words are clearly classified in the latter two dictionaries. It is generally accepted that the complex concepts of time in Mandarin Chinese are extended from these basic concepts of time.

As for glyphs of Chinese characters we choose, it is necessary to note that the evolution of the forms of Chinese characters has gone through the stages of Oracle Scripts, Bronze Scripts, Great Seal Scripts, Small Seal Scripts, Clerk Scripts, Cursive Hand Scripts and Regular Scripts, gradually simplified and evolving from the pictographs to today’s stroke characters (*cf.* Baidu Encyclopedia).^1^ We therefore focus on tracing the original meanings of Chinese time interval words from the first four types of ancient glyph structures.

## The cultural origins of event-based metonymic time conceptualization in Mandarin Chinese

Metonymy and metonymic thinking in this paper sticks to the classic definition in Cognitive Linguistics. But for time interval words, due to their ideographic nature of Chinese characters, almost each of them forms a mini discourse in itself. This is somewhat parallel to ideas of Janda (2011), p. 360 about word formation “*In word-formation, the source corresponds to the source word that the derivation is based on, the context for the metonymic relationship is the affix, and the target is the concept associated with the derived word.*” For each time interval word as an event metonymy, the source of time-interval-word metonymies in Mandarin Chinese corresponds to the words themselves, the context for the metonymic relationship consists of the radicals or stems, and the target is the time interval concept associated with such a word.

Therefore, the following part will be centered on the formative process to show how metonymy works in the formation of time interval words in a somewhat etymological sense.

### Philosophical thoughts

The traditional Chinese philosophical view of time originates from “the unity of heaven and man,” where time itself is regarded as the ontology, and human activities and time operate as a whole with mutual influence and function (He, 2010). Zhuangzi’s famous philosophical thoughts “if we live with the heaven and earth, we stay in harmony with everything (天地与我并生，万物与我合一)” (*Yi Zhuan • Classical Chinese*《易传·文言》) reflects the unity of time and space, as well as what there is and what happens in the world.

**Figure 1:**
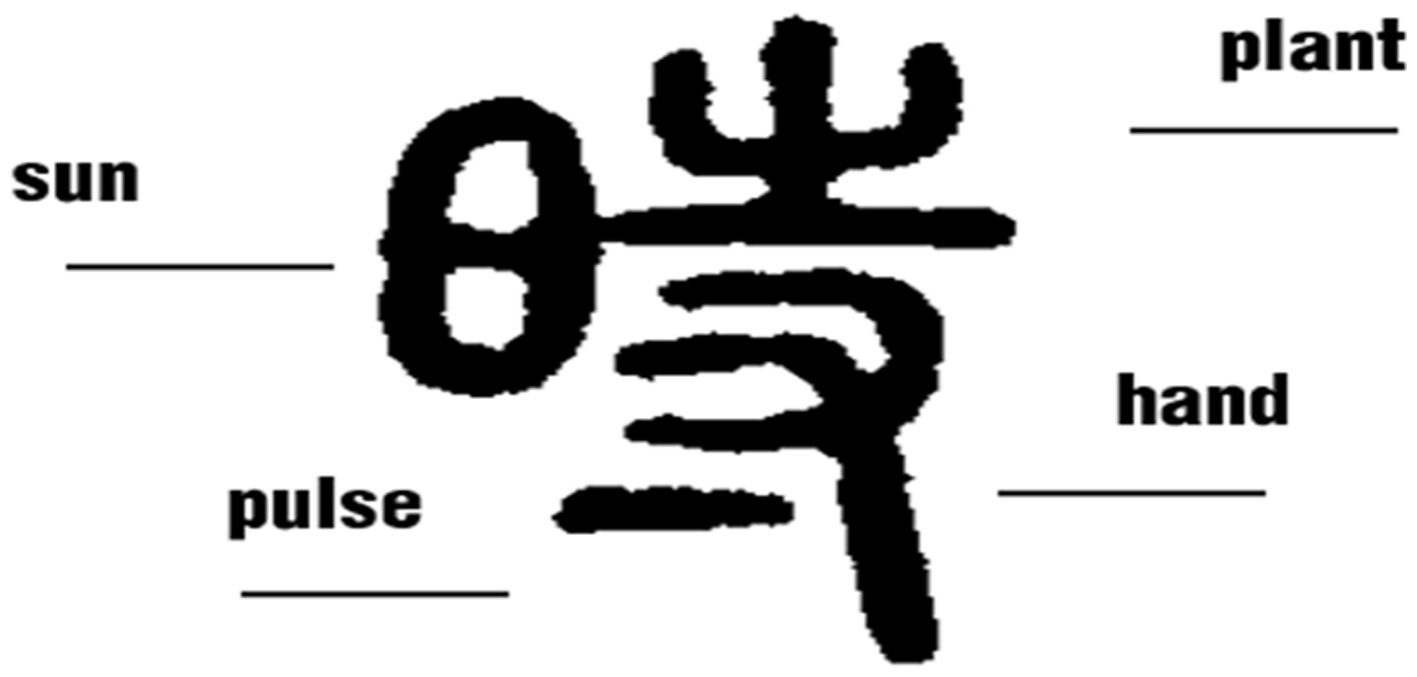
Glyphs of season [Small Seal Script].

One of the best examples of this philosophy is reflected in the Chinese character 时*shi*. In modern Chinese, 时*shi* mainly refers to 时间*shijian* (time) or小时*xiaoshi* (hour), but in ancient Chinese, the character was coined to represent the four seasons or the farming seasons throughout the year, and as can be seen in Figure 1, its glyph structure presents a clear portrayal of the philosophical idea of “the unity of heaven and man.” On the left-hand part of the character is the image of the sun, which is the major reference source for time conceptualization for the ancient Chinese, whose initial concept of time was based on the movement of the sun, while the right-hand part of the character can be further divided into two parts, with the upper part shaped like a plant and the lower part a human hand adjoined by a short line which indicates the human pulse. Nurtured by sunlight, the vibrant life of the growing plant and the pumping heart tie both nature and man in the alternation of the seasons since the two are considered as an integrated whole according to Chinese philosophy.

Immersed in the philosophy of the “unity of heaven and man,” Chinese ancestors *zu xian* [a Chinese word, which refers to the previous generation of a nation or family, especially the older generation, see also *Modern Chinese Dictionary (7th Edition)*, Dictionary Editorial Office of Institute of Linguistics, Chinese Academy of Social Sciences, 2016] regarded time as an innate feature of nature, and perceived the process of time through the laws of movement and the observable cycles of change for all natural things. Thus, “events” or “happenings” in the natural environment were adopted as their cognitive reference for their concepts of time. Based on this philosophical view of time, the Chinese language has evolved into the cognitive pattern of “temporal sequence reference first.” For example, the cognition of Chinese temporal orientation tends to be that earlier time is represented by上*shang* (up) or前*qian* (forward) and later time is represented by下*xia* (down) or *hou* (backward). In addition, the word order in Mandarin Chinese follows the principle of temporal sequence, in which events that occur first precede the ones that occur later. The structure of language directly reflects the temporal structure of reality, and the temporal sequence law of Chinese comes from observing and representing the real events, which is iconic in nature (Dai, 1988). This type of conceptual mapping, which occurs in the same cognitive domain based on relevance, adjacency or contiguity, reflects a kind of metonymic thinking, and thus it can be presumed that the deep philosophical bases of event metonymy in Mandarin Chinese embody the view of the “unity of heaven and man.”

### Yuzhou (universe): Double metonymic

Evidence for event-based metonymic time conceptualization in Mandarin Chinese can also be found in the ancient Chinese concept of “宇宙*yuzhou* (the universe).” In modern Chinese, this is a spatial concept, and a general term of all things in heaven and earth [*Ci Hai*《辞海》, Xia (1999)], while in ancient Chinese it refers to both space and time. Originally, 宇*yu* referred to the eaves of a house, meaning spatial extension, and 宙*zhou* represented the vertical poles and beams which support the house and determine its life span (Gao, 2014, p. 5). 宇 was then metonymically extended to denote the four cardinal directions (east, south, west and north) and 宙 denotes time (past, present, and future).

**Figure 2:**
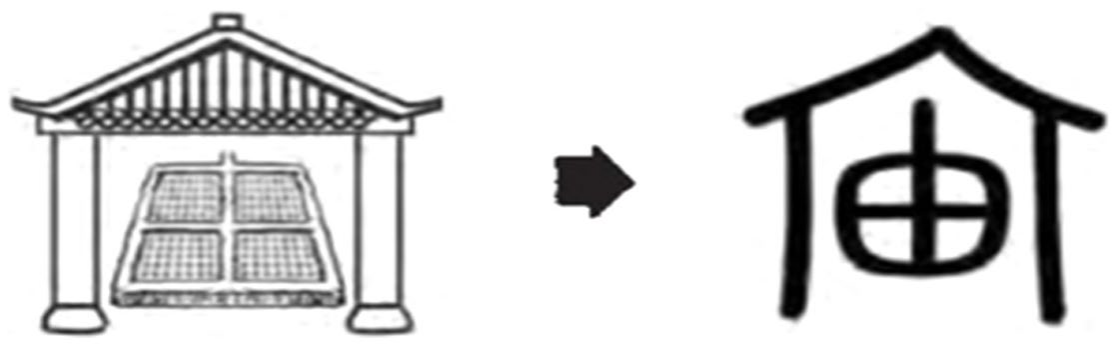
Glyphs of time [Bronze Script].

The way the character 宙*zhou* is formed also reflects the Chinese way of conceptualizing time in terms of events (see Figure 2). The upper part of the character is “宀,” which represents the image of the roof of a house, indicating that 宙 was originally associated with the house, and the lower part denotes the pronunciation of the character. “宙*zhou*,” as the beam of the house, bears the entire roof and is the backbone of the roof designed to withstand gravity. The quality of “宙*zhou*” is therefore related to the safety of the house, which is the guarantee for the house to maintain its structural integrity over time, and the guarantee that the ancestors and their descendants would live in peace and security. From this point of view, the existence of “宙” provides a good illustration of the passage of time (Liu et al., 2018). The character-making process of “宙” reflects the transformation from the object image and event image to the time image, and the temporal meaning of 宙 undergoes a double metonymic process. Here, at the first level, metonymy lies in the use of the vertical poles and beams to refer to the house, which are the interrelated parts comprising the whole and belong to the concept domain of “house.” Metonymy at the second level uses the existing state of the house to refer to the duration of time spent in that event status.

### The chronicle of timekeeping and evidence of “linguistic” representation in ancient artifacts

The embodiment of the concept of time held by the ancestors as depicted in the language system profoundly reflects the historical imprint of the Chinese nation’s way of understanding the world. In the philosophy of the Han nation, the nature of things reflects their position in time and space. The importance of events also corresponds to their positions in time and space. The events are arranged in order of priority, and those that come first are always given priority (Needham and Wang, 1956, p. 229). Long before written language appeared, the Chinese ancestors used knotted ropes to record what was happening around them. According to *Interpretations of Zhouyi* (《周易注》), “Knotting a rope is a covenant; if the event is of great importance, a big knot on the rope will be tied, and small knot for minor event.” The size of the knot represents the importance and priority of the event, which is probably the earliest “linguistic” representation of the concept of time. After that, the Chinese ancestors recorded events by engraving marks onto various artifacts such as wood, stones, bamboo strips and painted pottery. These carved symbols are regarded as the rudiments of Chinese characters, and their representation is no different from knotting cords. Later, the ancestors began to use drawings to depict objects related to the events that they wished to record, and the pictorial symbols gradually simplified and the pictographs evolved into hieroglyphs (Guo, 1972). Chinese archeologists have published a range of evidence in recent decades concerning the use of objects and drawings to record events, which provides evidence that abstract concepts represented by Chinese characters are essentially the result of metonymic conceptualization of events. It is worth noting that this kind of cultural artifacts, as time mark evidence for recording events, is of great significance across cultures. The research of Silva Sinha (2019) shows that knots and wood marking are also the basic concepts for time reckoning in indigenous Amazonian and Xinguan cultures and languages. Each of these cultures uses number-based cognitive artifacts in time reckoning: for example, there are knots in a string in Awetý and Kamaiurá, and marks on a piece of wood in Huni Kũ. Similarly, in the Inca civilization of the Andes many domains were measured by the quipu, a system using knots, including the famous pacha quipu, which was a kind of calendar, and a concept still in use in the Aymara and Quechua languages (Sinha and Gardenfors, 2015).

This method of time reckoning is essentially a way of representing the conceptualization of cognitive objects in terms of event metonymies. Chinese words for positioning and measuring time all reflect this cognitive approach, ranging from words for concrete time, such as year (年*nian，*岁*sui*), four seasons (*chun*, 夏*xia,* 秋*qiu*, 冬*dong*), month (月*yue*), day and night (*zhou，*夜*ye*), and different periods of the day (like 旦*dan* “day break,” 朝*zhao* “early morning,” 暮*mu* “*early evening*,” 昏*hun “*dusk”), to words for abstract time (like 期*qi* “appointed time,” 昔*xi “*past,” 畴*chou* “past; previous,” 顷*qing* “instant, past,” 霎*sha* “moment”) and general time (like 初*chu* “beginning,” 始*shi* “initial, start,” 古*gu* “ancient, old,” 今*jin* “the present,” 久*jiu* “long time”).

## Types of event-based metonymies in the conceptualization of time in Mandarin Chinese

Previous studies classified metonymies from two perspectives. One is the referential metonymies from pragmatic point of view proposed by Warren (2006), p. 5, which means relating one entity with another. The other is from the psychological point of view put forward by Slabakova et al. (2016) and Mario (2020), differentiating conventional or regular and novel metonymies (those that are “produced and comprehended online”; Slabakova et al., 2016, p.176). But the Cognitive Linguistic view holds that the experiential basis of metonymy conceptualization enjoys universality across cultural patterns and at the same time allows for cultural difference (Zhang et al., 2015, p. 220). This leads to the paper’s view that metonymy is both universal and cultural specific in some way; therefore, the way and the pattern of conceptualization of time in Chinese has its roots in Chinese culture, e.g., Chinese words related to a specific time interval all describe an event, but they may have different characteristics in the process of metonymy conceptualization. The paper outlines five major types of event metonymies functioning interactively in the following sections.

### Event image for the time of its appearance

When discussing the concepts of time, it is inevitable that the thinking mode of Chinese philosophy becomes involved in the discussion. Unlike western traditional way of thinking, which stresses names and concepts but discard images, the traditional Chinese way of thinking is the combination of all three. Numerous temporal terms, such as 年*nian* (year), 岁*sui* (age), 四时*si shi* (four seasons) and宙*zhou* (time), were initially all visual images, which then evolved into abstract concepts (Liu, 2000, p. 2).

An image is the result of human interaction with the world, and the mental (or physical) representation of something based on memory and imagination (Lakoff, 1987, p. 444). Its representation is the analog representation of concrete things, events, or activities, comprising the perceptual content represented in conceptual behavior (Liu and Li, 2018). All Chinese characters are ideographic to a varying degree, and are the product of viewing and reproducing images of an event or event-related object(s) from nature and society, expressing intuitiveness and symbolism (Zhu, 2016). This is clearly evidenced in the shape of the Naxi pictographs in the Sino-Tibetan language system and the oldest Chinese characters.

The ideographic nature of Chinese characters epitomizes the metonymic way of conceptualizing time according to the circadian rhythms and cycles of the sun and moon, which are the most intuitive observable objects. Basic time-interval words like 日*ri* (day), 昼*zhou* (daytime), 夜*ye* (night), 旦*dan* (day break), 朝*zhao* (early morning), 暮*mu* (*early evening*), 昏*hun* (dusk), 时*shi* (season) are all natural images based on physical recognition of the position changes of the sun and moon, which have strong cognitive prominence.

The original meaning of 昼 *zhou* is day or daytime, and according to *Shuo Wen Jie Zi* (Xu, 1963; henceforth referred to as “Shuowen”): “昼, is the opposite of night, and means brightness, indicating the rising and setting of the sun.” In Naxi pictographs, Oracle Scripts and Bronze Scripts, the glyphs of 昼 are simple pictograms of the sun (see Figure 2), meaning that when the sun rises, the daytime is coming or a new day begins. Chinese ancestors therefore referred to the time period from sunrise to sunset with reference to the image of the sun.

**Figure 3:**
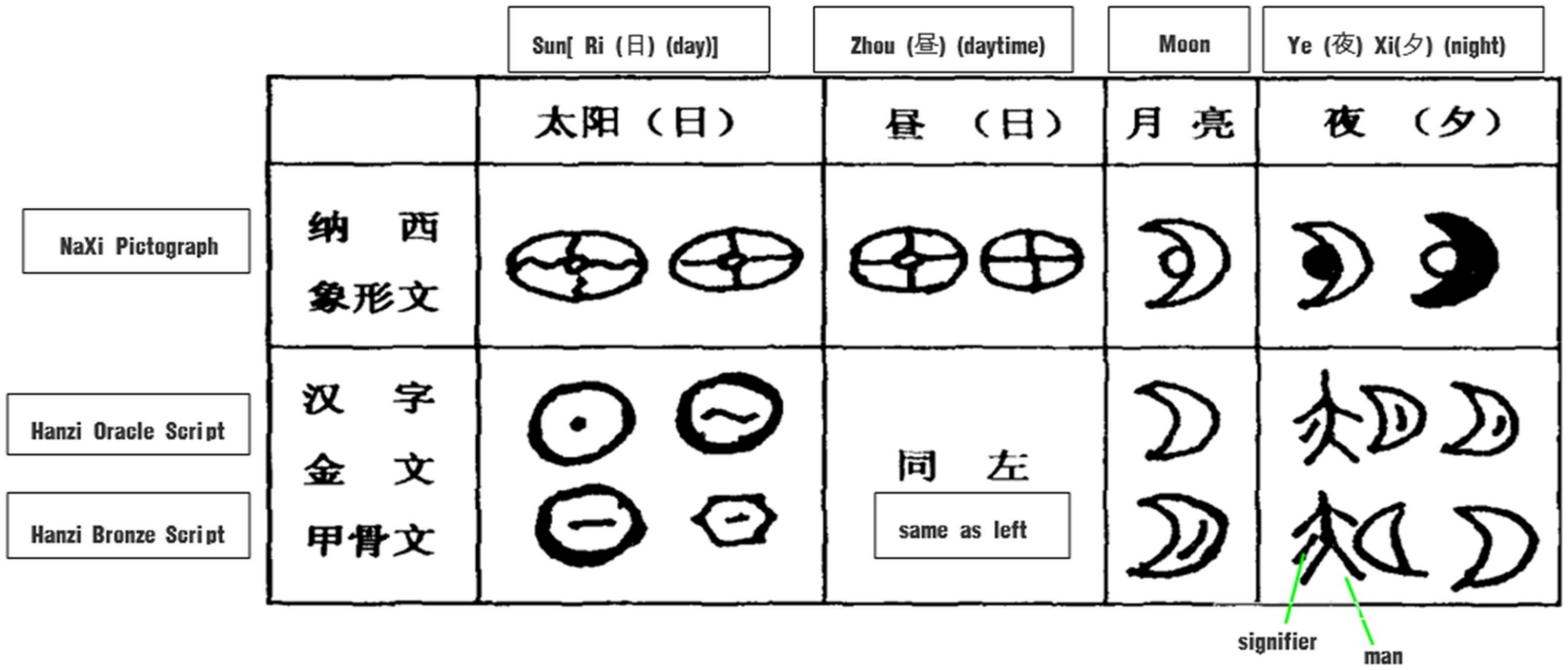
Glyphs of day and night.

Accordingly, the original meaning, 夜*ye* is night, and according to *Shuowen*: “夜 is the time when the world rests.” It is believed that 夜 means the time that people will stop their work in the terraced fields and walk home in order to rest. In Naxi pictographs, Oracle Scripts and Bronze Scripts, the glyphs of 夜 are the pictograms of the moon (see Figure 3), meaning that when the moon comes out, the night is coming. In other glyphs of Oracle Scripts and Bronze Scripts, the image is like a standing person, the left pointer is a signifier, indicating the area under the arm of a person, and on the right is the moon, indicating that it is night when the moon rises to the area under the arm of a person. It can be seen that the Chinese ancestors represented the time period from dark to dawn with reference to the image of the moon.

**Figure 4:**
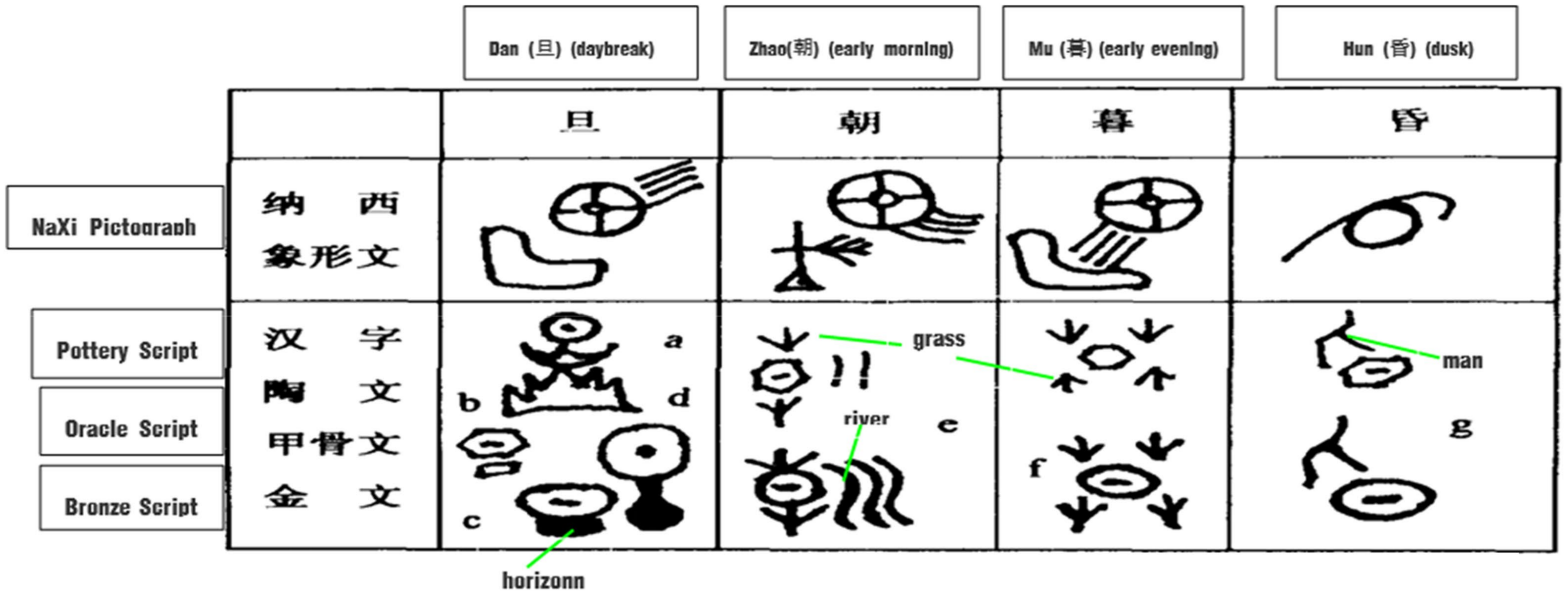
Glyphs of daybreak and evening.

The Chinese ancestors further distinguished between different time intervals in the day, such as “旦*dan* 朝*zhao* 暮*mu* 昏*hun*,” by the spatial position, motion or orientation of the sun and the changes of light and shade of the sky, as shown in Figure 4.

This point is best exemplified by the original meaning of旦*dan* meaning dawn. According to *Shuowen*: “旦 also means brightness.” In the Naxi pictographs and Oracle Scripts, the glyph is the appearance of sunlight on the horizon, while in Bronze Scripts this is the shape of the sun just rising but not yet leaving the ground. The ancient Chinese took the image of “the sun rising from the ground” to denote the time of daybreak.

The original meaning of 朝*zhao* refers to early morning. In Oracle Scripts, it is the image of the morning sun that has risen above the grass and trees, while in the Bronze Scripts, it is the appearance of the rising sun shining on the green grass by the river and the water rising with it. The ancients used the image of “sunrise among the grass and the river rising with it” to indicate the beginning of the day. 朝 can also be pronounced as *chao*, which is the original word for “𣶃/潮*chao* (tide),” suggesting the rising tide in the morning.

The original meaning of 暮*mu* is early evening or sunset. It was first written as “莫*mo*.” In Naxi pictographs, the shape of the character 暮 is the image of the sun which is descending toward the horizon, while in Oracle and Bronze Scripts, both the constructions are in the shape of four grasses above and below, with the character for the sun in the middle, indicating the interval of time toward evening when the sun is slanting westward and about to set into the grass.

Similarly, the original meaning of 昏*hun* is dusk. According to *Shuowen*: “昏 means darkness.” Both the Oracle and Bronze Scripts of 昏 show the sun has already set to a height within one’s reach. The image of “sunset in the west” indicates the period of time after sunset until it is completely dark.

From the above examples, it can be seen that the character-building process of Chinese time interval words can be regarded as a structured process of representing observed events or event states and captured images. Event image metonymies provide a pivot for the conceptualization of time, allowing the observer to enter and be in the scenario, scene or state of a concrete event in the natural environment, thus enabling a shift from the perception of common, specific events or event states to the abstract concept of time.

### Event schema for the time of its occurrence

As Kovecses (2005, p. 144) mentioned, our knowledge of the world comes in the form of structured frames, schemas, or ICMs. These can be construed as wholes with parts. Since frames are conceptualized as wholes that have parts, there are two general configurations of wholes and parts that give rise to metonymy-producing relationships: the “whole and its parts” configuration and the “part and part” configuration. A variety of specific metonymy-producing relationships can be observed within both configurations. Radden and Kovecses (1999) also claimed that events consist of both participants and relationships. The relationships within an event may include their purpose, cause, result, action, process, state, means and manners. Sinha and Gärdenfors (2015) further pointed out that compared with categories such as space, time, and objects, events may be more cognitively fundamental, and event schemas may play a more fundamental role in the conceptualization of time than spatial schemas, because people can understand and represent events more easily when based on their related elements. This means that a more notable or essential event schematic sense may evoke, and metonymically represent, the time of its occurrence.

**Figure 5:**
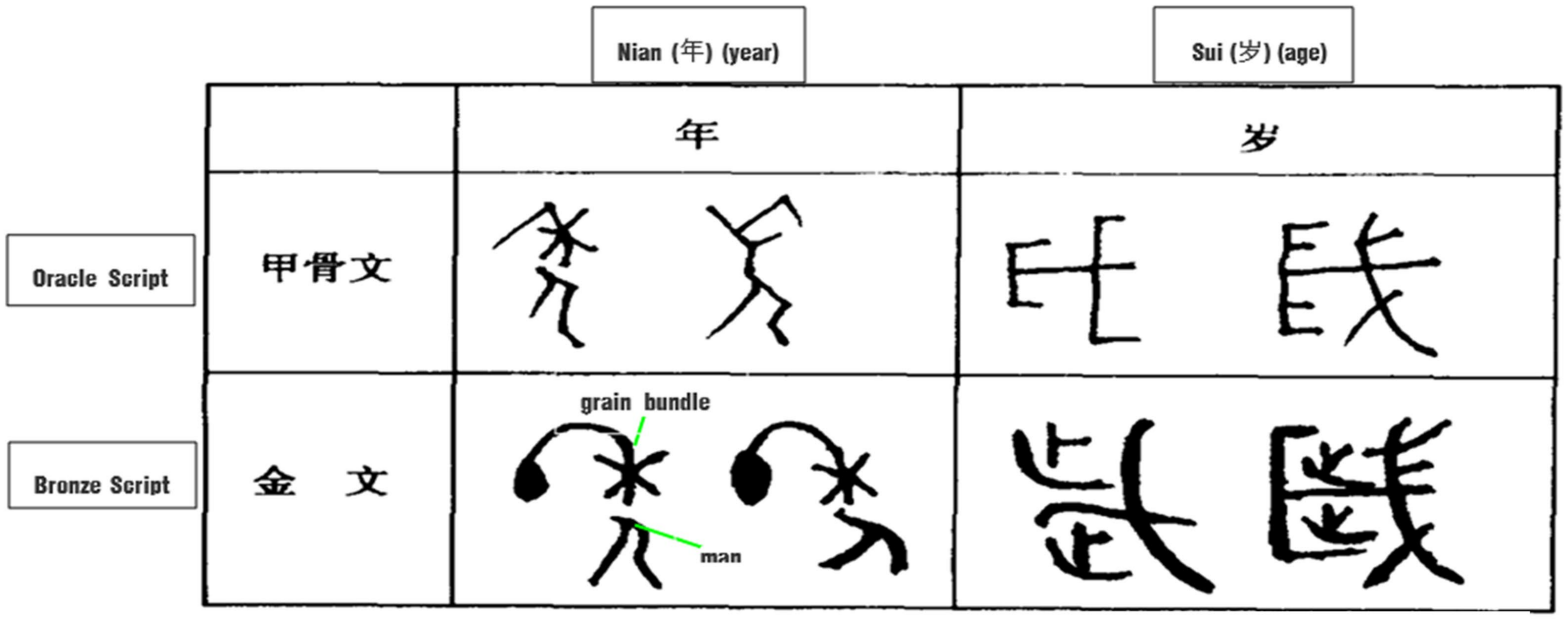
Glyphs of year and age.

Temporal interval words like “旦*dan* 朝*zhao* 暮*mu* 昏*hun*,” as explored in Section “Philosophical thoughts”, are both event image metonymies and event schema metonymies, which are intertwined in the cognitive process. 年*nian* (year) and 岁*sui* (age) from the Oracle and Bronzes Scripts (see Figure 5) are another two typical examples.

The character-making process of “年*nian*” can be understood as a double metonymic process. “年*nian* (year),” borrowed its pronunciation from 稔 (a kind of ripening crop), and originally meant “the ripeness of the harvest.” In Oracle and Bronze Scripts, the upper part of 年 was in the shape of a grain bundle, while the lower part represents the shape of a man bearing the grain bundle. The whole glyph presents a man trudging along with a bundle of grain on his back, metonymically standing for harvesting. The second metonymic process is the part-whole relationship of seasons and the year. In Chinese, there is a way of saying a year (*nian*) by just spring and autumn, which is a direct reflection of the agricultural civilization. More specifically, the concept of a year of agricultural production is used with reference to a complete process from sowing in spring to harvesting in autumn and the most important agricultural activities in these two seasons are then used to represent a year of agricultural activities.

Similarly, “岁*sui*,” borrowed its pronunciation from 穗 (a head of a wheat or rice plant), and originally meant “cut.” In Oracle Scripts, the Chinese character 岁 is in the shape of a large scythe with a curved blade. There are two dots on the axe head, which were originally ornaments, but later changed to 2 feet in Bronze Scripts. Since farmers harvest their crops once a year, the instruments used to harvest crops were borrowed to express the concept of time in terms of “year” or “age.” This is also a double metonymy, including the metonymy using the whole process of production in terms of means and results, and the last time node of agricultural production used as a metonymy for the whole time process.

According to *Guliang Zhuan · The 16th Year of Xuangong*(《谷梁传·宣剬十六年》), a year where all the crops yield a bumper harvest is called “大(有) 年*da (you) nian* (a big year), 乐岁*le sui* (a happy year), 富岁*fu sui* (a rich year), 丰年*feng nian* (an abundant year), 登年*deng nian* (a harvest year) or 岁定*sui ding* (a peaceful year).” A year where all the crops fail is called “小年*xiao nian* (a small year), 凶年*xiong nian* (a tough year), 岁凶*sui xiong* (a fierce year), 饥岁*ji sui* (a hungry year) or 荒年*huang nian* (a famine year). Chinese people are still fond of using the term “望岁*wang sui* (looking forward to a good year)” to express their expectation of a good harvest. These terms reflect the inevitable connection between agricultural production and social stability in China.

It can be seen that the original meaning and the glyphs of “年” and “岁” indicate that most of the early Chinese agricultural communities recorded time by farming activities with reference to the cycle of crops from ploughing to ripening and lastly to the harvest by means of sub-events or tools in the harvest event schema.

### Typical subevent occurrence for the time

Peirsman and Geeraerts (2006), p. 290 argue that the prototypical structure of the domain of actions, events and processes again has its core in PART FOR WHOLE relationships between bounded entities. Instead of relating two spatial entities or two periods of time, however, the metonymical pattern SUBEVENT FOR EVENT comprises two actions, events or processes, one of which is conceptualized as a part of the other. This metonymical pattern thus allows us to pick out one (or more) sub-event(s) of the overall/complex event in order to refer to this overall/complex event. As events have a multifaceted nature and surrounding elements like aspect, time, modality, grounding, and setting, all which may serve as metonymic reference points for evoking the Event ICM (Idealized Cognitive Model) as a whole to conceptualize time (Radden, 2013). The concept of time held by the ancient Chinese also reflects the characteristics of their abstract conceptualization of the most typical and striking sub-event(s) in nature and daily life.

**Figure 6:**
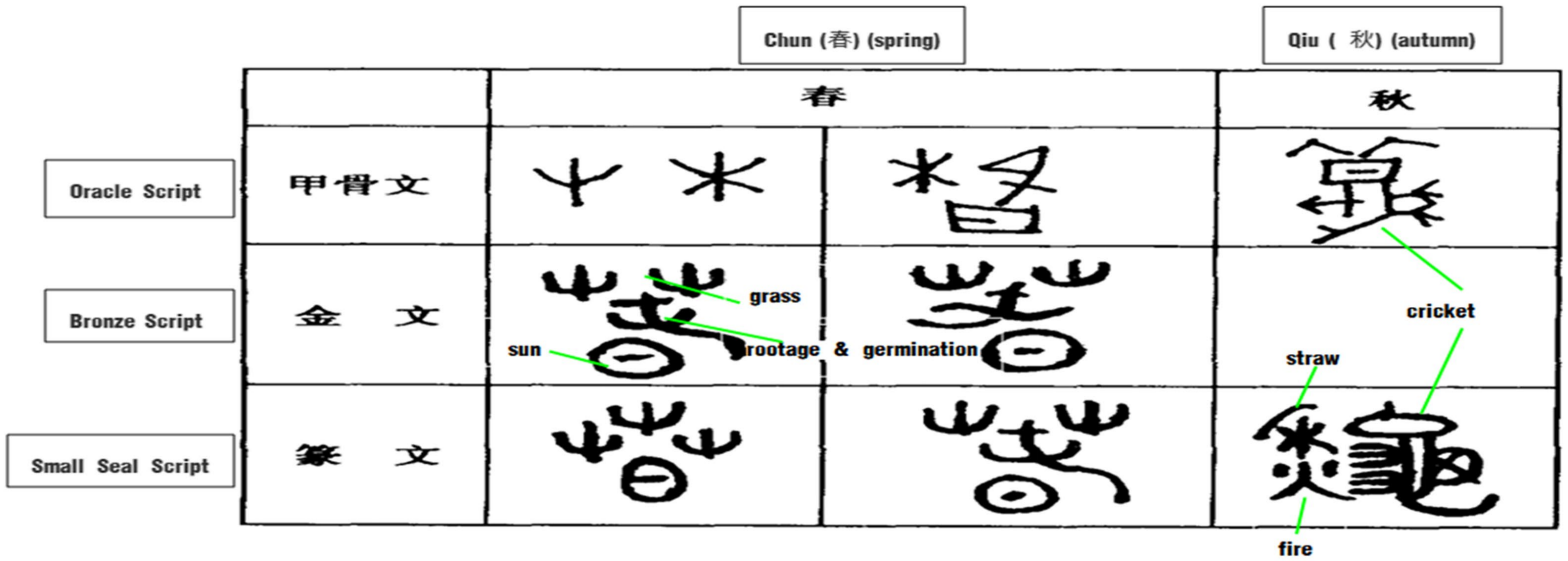
Glyphs of spring and autumn.

In ancient agricultural times, sowing and harvesting were the two most important events in the agricultural activities of the year. The cycle of sowing in spring and harvesting in autumn not only marked the beginning and end of their agricultural cycle, but also represents the life cycle of plant growth. The configuration of the character “春*chun*” reflects the long germination of grass as the most prominent feature of these sowing events, while the construction of “秋*qiu*” embodies representative scenes and events of autumn (see Figure 6).

“春*chun*” original meant the sprouting of grass. According to *Shuowen*: “Spring is a season when Yang energy (the positive force in the universe) is initiated and starts to rise, so it promotes the sprouting of all things.” Besides, it is also recorded in *The Book of Han—Laws and Calendars* (《汉书·律历志》): “Spring is also the season of birth and growth, grass and trees sprout and everything comes back to life.” These two sentences summarize the seasonal characteristics of spring, when grasses and trees are budding and everything is flourishing. Scripts from the Oracle bones, Bronzes and even Small Seals all show that the character “春” is composed of the shape of grass, the sun and “屯*tun*,” and in accordance with *Shuowen*’s explanation, the Chinese character “屯*tun*” is like the first sprouts of vegetation peeping forth, portraying an image of grass seeds sprouting. Thus, the whole Chinese character “春” means that the sun enables plants to sprout.

“秋*qiu*” original means the ripening of the crops. As explained by S*huowen*: “Autumn is the time when the grain is ripe.” As Duan (1981) explained (see also *Notes to Shuo Wen Jie Zi*): “At this time everything is full-grown, and nothing is more valuable than the grain, thus it is selected as its stroke.” In Oracle scripts, the character for “秋” is in the shape of a cricket. The Chinese ancestors discovered that insects with long feelers or antennae, such as crickets, usually chirp in the autumn, so they borrowed the shape and sound of crickets to convey the meaning of “insects chirping in the autumn” when creating the character for the seasonal time concept “秋*qiu*.” As can be seen, in Small Seal Scripts, the upper left part of “秋” is in the shape of straw with the addition of a character “火*huo* (fire)” under the cricket shape, which means that after harvesting crops, the straw is then burned to eliminate the pests and the ashes are to be used as fertilizer for the soil. Sowing and harvesting, chirping crickets in autumn, and burning stubble to exterminate pests for the following year are all important considerations in agricultural production. As can be seen from the analysis, “秋*qiu*” as a time interval word has undergone triple metonymic processes about the events involved.

The whole process of productive work for a year starts with the sowing of seeds and ends with the burning of stubble, constituting a continuous cycle. Therefore, “春秋*chunqiu* (spring and autumn)” is often used to express “年*nian* (year)” in Chinese. According to the Baidu Encyclopedia,^2^ the cycle of sowing and harvesting represents the cycle of time, which led ancient scholars to believe that the cycle of spring sowing and autumn harvesting represents the life history of the Chinese people. This is why the Chinese history book was named “*Spring and Autumn*” (《春秋》).

The Chinese characters for time interval words were therefore created by borrowing their constructional features and ideographic ways to express the landmark events of that period of time, reflecting a high degree of integration between the ancestors’ concept of seasons and seasonal characteristics.

### Manner of the event for the time of its occurrence

China was, and still is, a large country with many ethnic groups all with different cognitive perspectives on the process of conceptualizing time due to differences in their living environment, production methods and lifestyles.

It is important to note that although words related to specific intervals of time are coined based on the metonymic conceptualization of corresponding temporal events. In the traditional Chinese view of time, however, the concepts of “event time’ and “opportune moment” or “kairotic time” coexist (Kairotic time^*^*^2^ was explored by Birth, (2012), and he mainly discussed this in relation to ancient Greece). This is particularly noticeable in the metonymic conceptualization of time in terms of event mode. A distinctive feature of the traditional Chinese view of time is that it is closely linked with the process of farming and the rhythms of nature, and because farming is most concerned with the timing of the seasons, the ancient Chinese believed that agricultural activities should be undertaken in accordance with the “opportune time.” For example, “時*shi* (four seasons or the farming seasons throughout the year)” originally meant “sowing” in the early Qin Dynasty, but was later known as “莳*shi*.”

The 24 Solar Terms are a system based on a knowledge of time created by the Chinese to observe heaven and earth and learn about the natural world. They are also the basic guidelines for undertaking agricultural production and coordinating farming activities, representing the Chinese philosophy of life in a society that follows the “opportune moments” that guides practice.

In addition, the *YiJing*, or *Book of Changes* (《易经》), is the first of the Five Confucian Classics in China and can be seen as the wellspring of both Confucianism and Taoism. To some extent, *YiJing* can represent the main ancient Chinese traditional culture. *YiJing* is written with examples from the daily life in the ancient period; not only ordinary events happening every day, but also special events like rituals, war, various celestial movements and natural phenomenon like solar eclipse, which are closely related to the original conceptualization of time in ancient China (Yang, 2020). *YiJing* is also a classical philosophical book which promotes the proposition that “people should conform to nature and act at the right time,” and the “propitious or opportune time,” mentioned many times in the book, which is the notion of time as the right or proper time to do something, as it is generally believed that engaging in certain activities at this right time will bring good luck. This sense of “timely chance” began to be presented in the time consciousness of Chinese ancestors in the Zhou Dynasty, who firmly believed that the so-called “timely chance” was of great importance in their natural or agricultural world. The practice of “divination” in the book certainly also evidences how the concept of “opportunity time” was extremely important.

With regard to the temporal concept of “年*nian* (year)” and “岁*sui* (age),” the Chinese Han ethnic group, with a primarily farming culture, recorded the new year by the ripening of grain (see Figure 5), while the Tibetans mark the beginning of the year by the ripening of wheat. Among some remote ethnic minorities, the custom of reckoning the new year by phenology is still preserved. For example, the ethnic group of Gaoshan in Taiwan takes “the ripeness of millet” or “the blossom of erythrina” as the start of a new year, while other ethnic groups in Yunnan regard “the call of the cuckoo,” “the blossom of *Bombax ceiba*” or “the ripeness of rice” as the passing of a year (Liu, 2006). Northern nomadic and hunting communities, even up to the time of Genghis Khan (1,162–1,227) of the Mongol nation, counted the year according to the time at which the grasses turn green, and when they refer to their own age 岁*sui* (*age*), they recall how many times they have seen the grasses turn green. Ethnic groups in Yunnan province, like the Nu ethnic group (怒族), Dulong ethnic group (独龙族) and Lisu ethnic group (傈僳族) conceptualize the time of year by planting a palm tree (董棕树) in front of their houses, which usually matures and bears fruit after 30 years. After that, another such tree is planted (Liu, 2000). In addition, the Hezhe people in the lower reaches of Songhua River who depend on fishing record their age by “the number of times they have eaten salmon” referring to the annual salmon spawning run, while the Yamei people in Taiwan count the year by “the annual return of the flying fish” (Hao and Chen, 2004, p. 65).

With respect to the temporal concept of “季*ji* (season),” the character of which is composed of “禾*he* (standing grain) and “子*zi* (son), indicating that it is a young crop. The rhythms regarding the division of seasons varies among different ancient Chinese ethnic groups depending on their respective lifestyles. The Han ethnic group, who mainly depend on agricultural production, took farming and plant growth cycles as their reference. According to the study of Oracle bone inscriptions, a year in Shang Dynasty was divided into only two seasons, namely 春*chun* (spring) and 秋*qiu* (autumn; see Figure 6), representing the planting and harvest of crops. The Chinese idiom of “春生夏长，秋收冬藏” can be interpreted as “all things sprout in spring, grow in summer, are harvested in autumn and stored in winter,” which describes what the Chinese ancestors thought was the “opportune moments” of the farming year, constituting a natural law which human beings could not disobey. In the Zhou Dynasty, to accommodate the further development of agricultural production, the ancestors divided the year into four seasons based on the growing cycle of grain. Naturally, nomadic people who lived by animal husbandry distinguished seasons by the color and prosperity of their pasture. For example, the Mongolian herders divided a year into four seasons according to the color, the ripening and the withering of the grass, but some herders who inhabited in the Al Horqin grassland divided a year into three seasons (冬春*dongchun “*winter–spring,” 夏*xia* “summer,” 秋*qiu* “autumn”) or two seasons (冬春*dongchun* “winter–spring,” 夏秋*xiaqiu* “summer–autumn”). The Oroqen people in Heilongjiang province also divided the year into four seasons based on the regular changes of snowfall, pasture and deer activities.

Hunting communities mainly distinguish seasons according to animal breeding cycles and migration activities. It is recorded that the Wuhuan people “distinguish four seasons through the pregnancy and lactation of birds and beasts” (*The Book of the Later Han Dynasty: Wuhuan Biography*《后汉书·乌桓传》), and the Li ethnic group in Hainan “keep track of spring and autumn by observing the birth of birds and beasts” (*Peace and Harmony • Danzhou Customs*《太平寰宇记·澹州风俗》).

**Figure 7:**
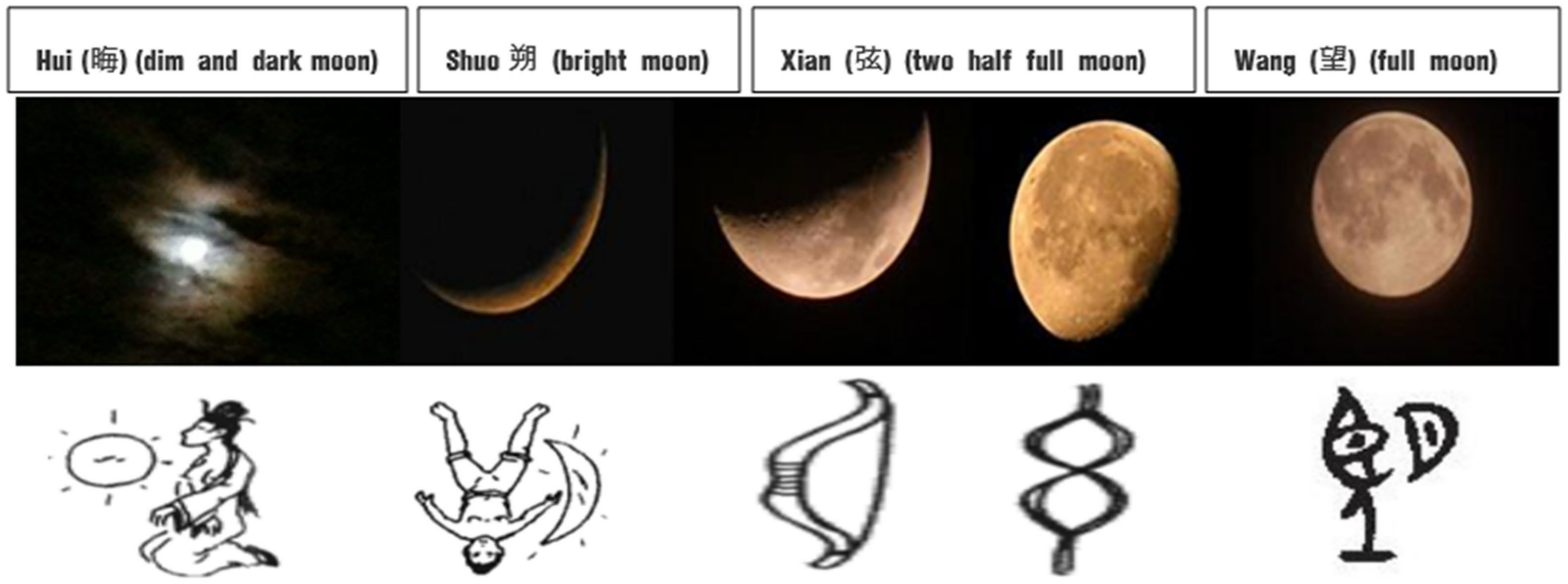
Glyphs of phases of the moon [Oracle Script and Bronze Script].

Ancient Chinese usually distinguished the temporal concept of “月*yue* (month),” with respect to their own life rhythm, farming time and phenology. For instance, the Lisu ethnic group (傈僳族) in Yunnan still use a natural calendar that divides the year into 10 “seasonal months,” each with a different length. These time interval words occur in the following order: New Year Month (January), House Building Months (February), Flower Blooming Month (March), Birds Singing Month (April), Mountain Burning Month (May), Hungry Month (June), Gathering Month (July and August), Harvest Month (September and October), Wine Boiling Month (November) and Hunting Month (December). The ancient Chinese also reckoned certain day(s) within a month based on their observation of the moon’s presence or absence, or waxing or waning in the sky. The pictographic characters like 晦*hui* (“dim and dark moon” indicates the end of the lunar month), 朔*shuo* (“bright moon” indicates the first day of the lunar month), 弦*xian* (“half full moon” indicates the seventh and eighth days of the first half of the lunar month), and the 24th and 25th days of the second half of the lunar month) and 望*wang* (“full moon” indicates the fifteenth day of the lunar month) are the most notable examples (see Figure 7).

The Chinese ancestors’ temporal concept of “日*ri* (day),” divided a day into 12 time intervals according to their daily production activities and living habits, combined with the natural laws of the sun and the changes in the light and darkness of the sky, and the laws of animal activities. These characters include: 夜半*yeban* (midnight, referring to the middle of the natural phenomenon change from dark to dawn, 23:00 p.m. to 1:00 a.m.), 鸡鸣*jiming* (cock crowing, 1:00 a.m. to 3:00 a.m.), 平旦*pingdan* (at the turn of night and day, 3: 00 a.m. to 5: 00 a.m.), 日出*richu* (sunrise, when the sun is just showing its face and rising, 5:00 a.m. to 7:00 a.m.), 食时*shishi* (breakfast time, 7:00 a.m. to 9:00 a.m.), 隅中*yuzhong* (when the sun has not yet reached its zenith, 9:00 a.m. to11:00 a.m.), 日中*rizhong* (when the sun travels toward the middle of the sky, 11:00 a.m. to13:00 p.m.), 日昳*ridie* (when the sun has just lowered toward the west from its zenith, 13:00 p.m. to15:00 p.m.), 晡时*bushi* (the second meal time, 15:00 p.m. to17:00 p.m.), 日入*riru* (when the sun goes down, it is also the time when the ancestors took a nap in the fields, 17: 00 p.m. to 19: 00 p.m.), 黄昏*huanghun* (when the sun has set and the sky becomes dark, 19:00 p.m. to 21:00 p.m.), 人定*rending* (when the ancestors stopped their activities and rested and slept, 21: 00 p.m. to 23: 00 p.m.). All these temporal concepts for hour names are related to the timing of events, and are strong evidence that the ancient Chinese conceptualized time in terms of events, and further demonstrates that the Chinese language has an empirical structure.

It can be seen that the concepts of time in the domains of life stages, seasons, months and the different times of day and night are closely related to the living environment, social life and methods of food production employed by the Chinese ancestors. Over the years, the ancestors positioned and reckoned time based on various rhythmic events and their presentation modes, such as the movement of celestial bodies, phenological changes, and farming activities combined with the rules governing social life and customs. To sum up, the metonymic conceptualization of time in terms of manner of the event in Mandarin Chinese essentially maps the intrinsic correlation and consistency between the modes of human survival and their cognition.

### Space–time conflation for the event occurrence time

The basic rules of human writing generally follow the linear principle from left to right. English written language is written in horizontal rows from left to right, while Chinese characters are written and read in vertical columns.

In addition, Meng (2014, p. 17) argues that the constructions of individual Chinese characters are endowed with dual attributes: linear time coding (in positioning the stroke order and the radicals) and nonlinear image coding (space). In spatial image coding, one of the key principles is that the spatial layout of the image elements is a copy of reality. As the layout of the radicals of Chinese characters is a copy of reality, it is also governed by nonlinear spatial law. Or, more specifically, the formation of Chinese characters normally abides by the linearity principle, but the positioning of the radicals has a great impact on their resultative meaning. Hence, as a result of the encoding process of Chinese characters, the time-interval words often exist in the form of space–time conflation metonymies.

**Figure 8:**
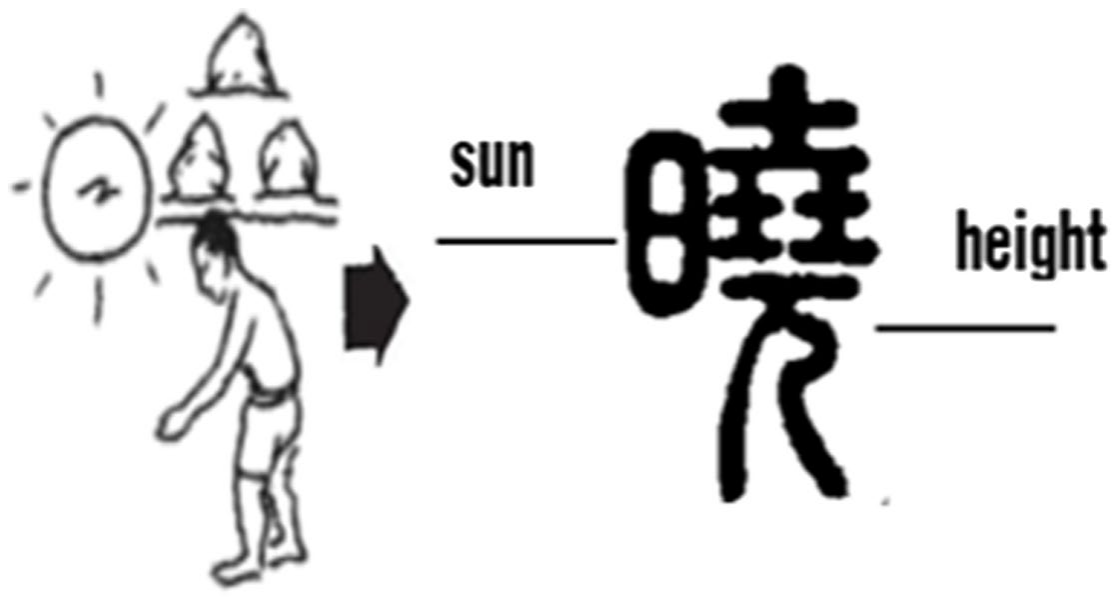
Glyphs of dawn [Small Seal Script].

For example, in the radical component “日*ri* (sun),” the different positions of “日” in the following Chinese characters imply different meanings (Liu and Xu, 2019): The morphological structure of these characters is endowed with dual attributes of linear time coding and nonlinear spatial coding. When placed on the left or the top of the character, it generally indicates morning or an early time of the day or evening or late time of the day, but when placed on the right or lower part of the character it represents past time. For example:

“晓*xiao*” originally meant “dawn.” According to *Shuowen*: “晓means brightness.” “日” on the left side of the character here indicates the sun, and 堯(尧) *yao* on the right side denotes both the pronunciation and “high” (see Figure 8), together, they indicate the time interval when the sun gradually rises higher.

**Figure 9:**
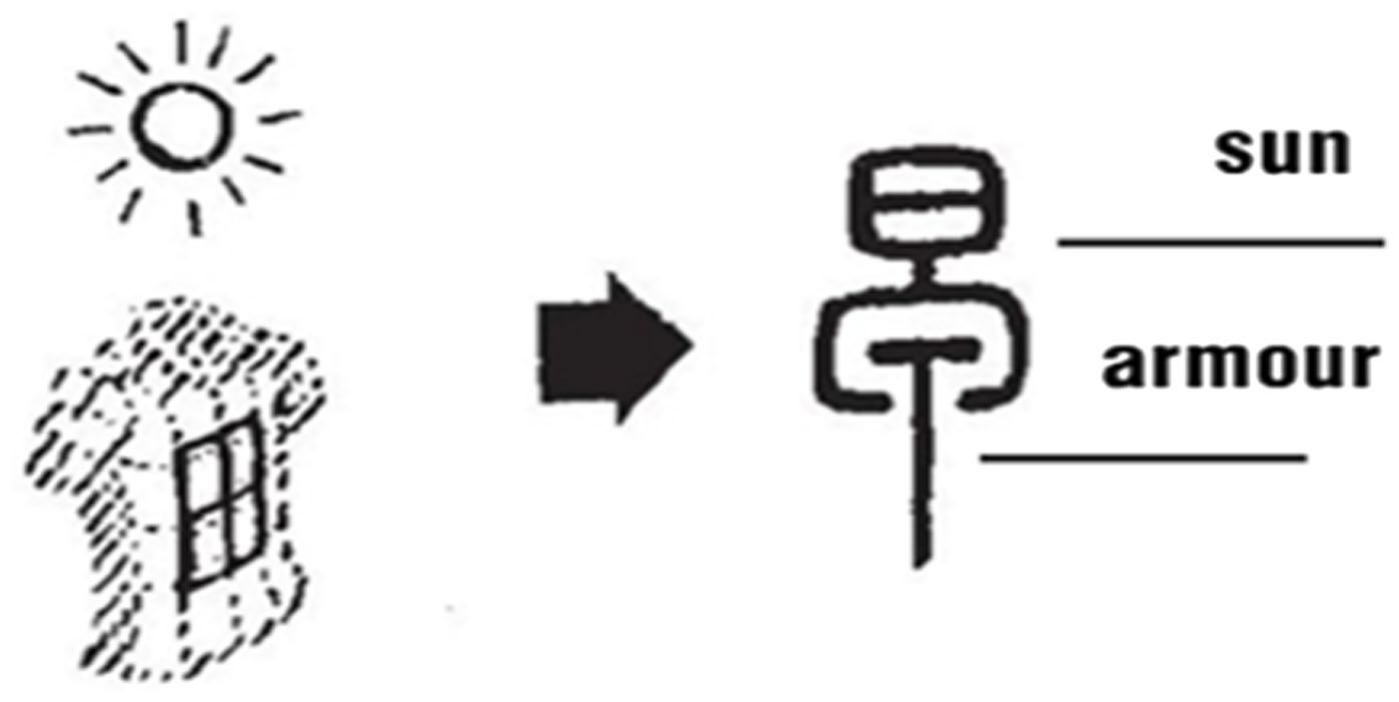
Glyphs of early morning [Small Seal Script].

“早*zao*” originally meant “early morning.” According to *Shuowen*: “早 means 晨*chen*.” The upper part of the character is also the sun and the lower part is the shape of armor (see Figure 9), and the whole character means that when the sun shines on the armor, the soldiers get up and start their morning training.

**Figure 10:**
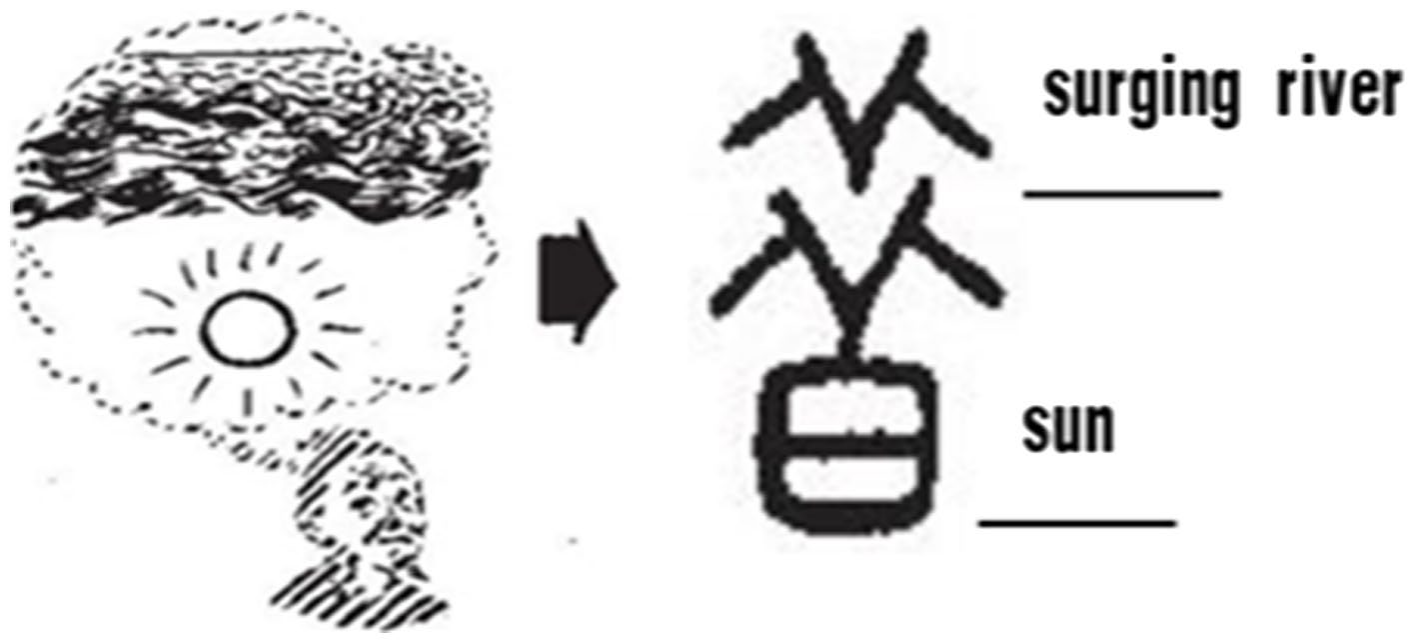
Glyphs of past [Small Seal Script].

“昔*xi*” originally meant “previously or past.” The upper part of the character looks like a river with raging waters (see Figure 10), and the lower part of the character is the sun. The combination indicates that the sun has fallen beneath the turbulent waters of the river, which means that even though the flood has passed, one must not forget the disaster it has wrought (Flooding was a common event in ancient times and was fresh in the ancestors’ minds). Thus, the way in which time is conceptualized as past depends on whether the event has already occurred.

In addition to “日*ri* (sun),” other radicals such as “月*yue* (moon)” and “星*xing* (stars)” have also been incorporated as part of the popular constituents of the time interval words in modern Chinese. One reason is that the ancient Chinese reckoned time not only by the position or shadow of the sun, but also by the movement of the moon and stars or the shape and configuration of the moon (Wang, 2004).

**Figure 11:**
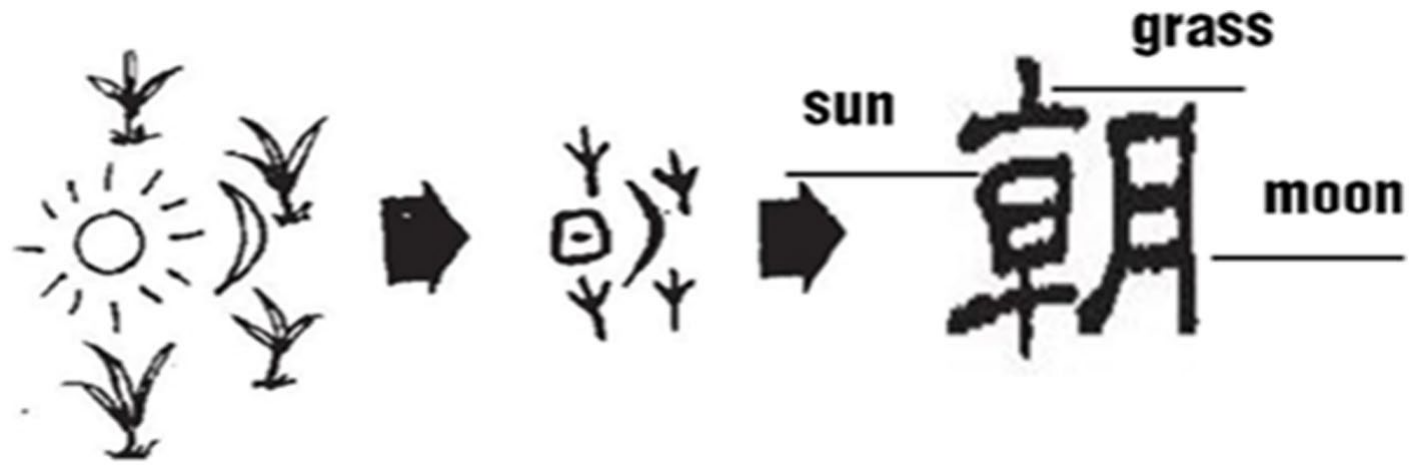
Glyphs of early morning [Oracle Script and Li Script].

For example, the character “朝*zhao*” mentioned above has many variations when incorporated on the right side of a character. In Bronze Scripts it is water-shaped, indicating that the river rises with the sun (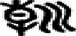), while in Small Seal Scripts it is boat-shaped, indicating that a rising tide lifts the boat (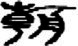), but its earliest glyph in Oracle Scripts is moon-shaped (see Figure 11), which best reflects the original meaning of the character, indicating that the sun shines on the green grass by the river, while the moon is still visible. The image of the event therefore indicates that “the sun and the moon shine together” which is borrowed to express the time interval of early morning.

**Figure 12:**
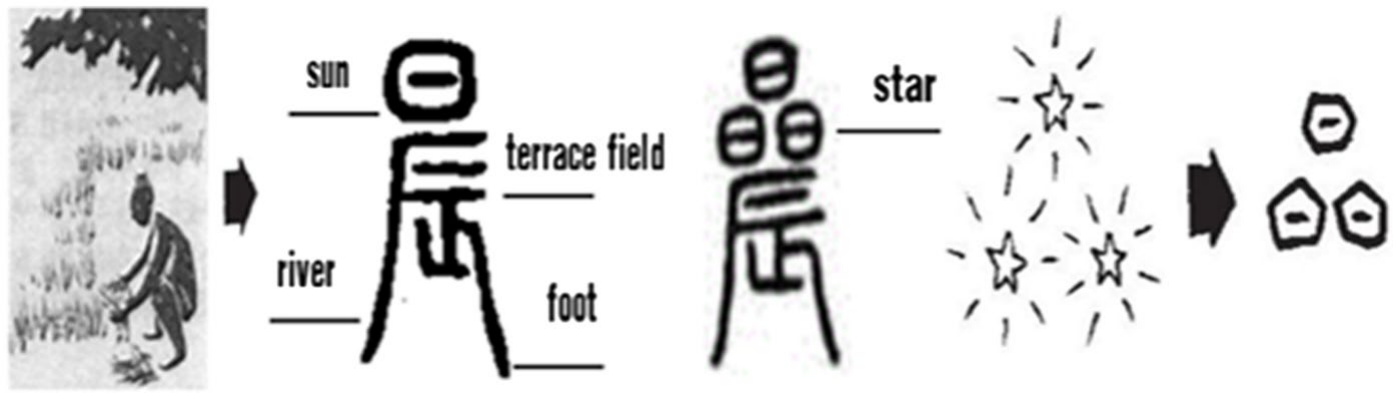
Glyphs of morning [Small Seal Script].

The original meaning of “晨c*hen*” is to cultivate crops. “晨*chen*” and “农*nong* (farming)” have the same origin in Oracle Scripts. The character is shaped like a farming tool held in both hands (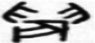), this is because the ancestors began to cultivate and work in the early morning, thus the character was created by taking the image of starting working with farm tools in hands to indicate “early morning.” In addition, there are two ways to write the character “晨” in Small Seal Scripts (see Figure 12), the upper part of which is the sun or the stars, while the lower part comprises the farming tool, for the stars are still faintly visible in the early morning when the ancestors begin to work in the fields by the river. The metonymic character-making process of 晨 perfectly demonstrates the diurnal rhythms -- “the alternation of the sun (*day*) and the stars (*night*),” therefore, in Chinese, the character 星*xing* (stars) means not only “*evening*” but also *“early morning.”* Even today, Chinese people still use “月落星沉*yue luo xing chen* (the moon sets and the stars sink)” to indicate the time when dawn is approaching, and “披星戴月*pi xing dai yue* (wearing stars and the moon)” to denote hard work from morning till night.

The radical position of the above-mentioned time-interval words can therefore be seen to affect the overall meaning of the Chinese characters. This has two epistemological implications: it reflects the Chinese ancestors’ understanding of the dialectical unity of time and space, where there is ‘a bit of me in you and a bit of you in me’ and also the sublimation of the level of cognition. Secondly, it embodies the characteristics of holistic thinking or the epistemology of the Chinese people, who were adept at grasping the fundamental features and internal logic of things within the context of intertwined relationships.

## Cognitive and cultural functions of Chinese time interval words as representational symbols

The linguistic representation of time conceptualization by time interval words in Chinese, rooted in Chinese people’s living environment, life style and their way of understanding the world, have their unique cognitive and cultural functions.

### The cognitive functions of time interval words

As one of the oldest scripts in the world, Chinese characters serve as an intermediary for human cognitive processes. Chinese characters are ideographic characters using a pictographic symbol system to record various events or event states, which is the result of the embodied philosophical thinking of “taking the bodies from near and comparing it to things from far” of the Chinese ancestors. It can be said that Chinese characters are pictographs that express their unique meanings or concepts by taking the essential features of the shape, interest and charm from ‘everything in heaven and earth’. Each character has a unique cognitive function which is the summation of the wisdom of the ancient Chinese ancestors. Through the origin and evolution of each Chinese character, not only can the internal relationship between the shape and meaning of each character be seen, but the way in which the ancestors lived, and how that life changed can be deduced, and even their thought processes can be revealed.

**Figure 13:**
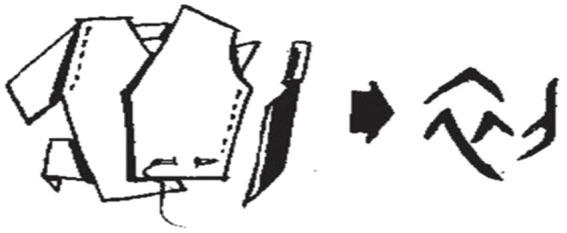
Glyphs of start time [Oracle Script].

In addition to the event-like time interval words mentioned above, the behavior-like time interval word “初*chu*” is another vivid example of cognitive function (see Figure 13). The original meaning of “初” refers to cutting clothes with a knife. In Oracle Scripts, the left and right sides of the character 初 are the shapes of a garment and a knife, respectively. The Chinese ancestors’ use of animal skins to ‘cover their shame’ and keep warm marked the beginning of human civilization, and the use of knives enabled the beginning of clothing making, so they took this iconic act to refer to the time concept of “beginning,” and it is evident that this method of character making greatly saved their cognitive resources.

### The cultural functions of time interval words

The form, sound and meaning of Chinese characters are rich in cultural factors, as they are the result of replication and development over time, and the influence of the national culture. The pictograph, as the foundation for the formation of Chinese characters, is the most basic practice of the national creative cultural spirit (Zhang, 2004).

When creating the time interval words, the Chinese ancestors did not randomly incorporate their own observations and experiences of the world into them, but gradually accumulated and condensed them under the strict guidance of national cultural psychology and modes of thinking. Chinese time interval words have evolved through an integrated relationship with social and cultural systems over time. Each time interval word contains a representation of the larger world and social history, and through the constructions of these words, information concerning ancient Chinese systems and practices such as time measurement, agricultural production, divination and sacrifice, and the astronomical calendar, can be deduced, allowing a peek into all aspects of the social lives of the Chinese ancestors. It is reasonable to say that Chinese time interval words not only enrich the vocabulary of Chinese language and highlight the systematic nature of Chinese characters, but also record and promote the development of Chinese culture.

**Figure 14:**
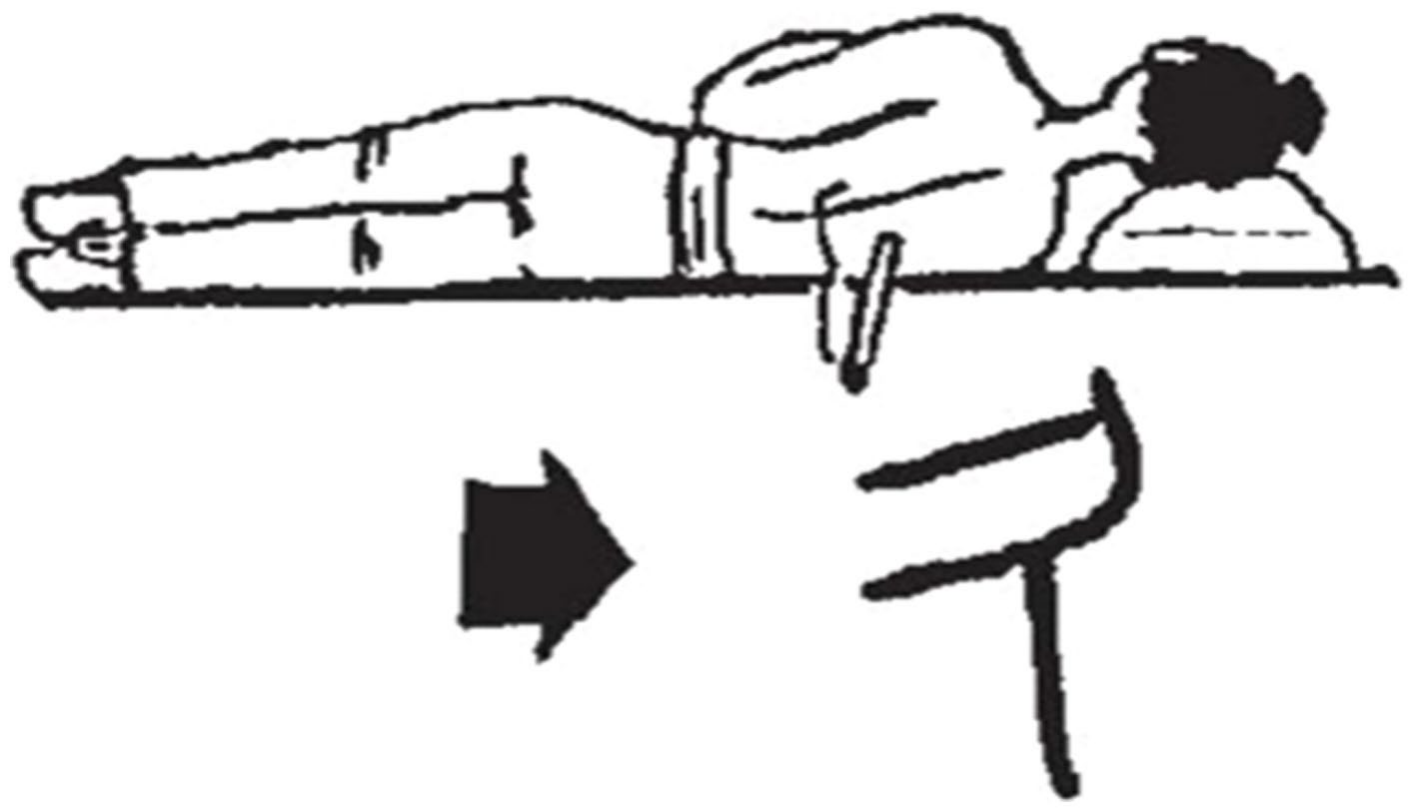
Glyphs of long time [Small Seal Script].

In addition to the various examples discussed above, another character 久 *jiu* (long time) is chosen to demonstrate the unique cultural function of Chinese characters (see Figure 14). The original meaning of “久” is “灸 moxibustion” (a natural therapy where a substance, moxa, is burned in close proximity to a part of the body to promote healing, or needles inserted to stimulate specific acupuncture points to regulate or correct the flow of “qi” to restore health, embodying the traditional Chinese medicine concept of “the unity of heaven and man”), which is a typical pictographic character, and its pronunciation is the same as “灸.” The glyph shows a person lying on their side with a therapeutic moxa stick (or a therapeutic needle) protruding from their back. Recognizing that the therapeutic effect of moxibustion was not an overnight process, the Chinese ancestors captured the image of moxibustion or acupuncture to represent the temporal concept of a “long time.” It can be seen that the shape, sound and meaning of 久 all explain the spirit of the traditional Chinese medical culture.

To sum up, Chinese characters are self-originated characters created without any external influences. The creation of Chinese time interval words is a unique aspect of ancient Chinese characters and authentic native culture. Each time a time interval word is generated based on the metonymic conceptualization of an event it has its own unique cognitive and cultural interpretative functions. Chinese time interval words are therefore strong evidence of the linguistic representation of time conceptualization in Mandarin Chinese. It is important to note that the glyphs and original meanings of these time interval words have necessarily had a significant impact on the Chinese understanding of the concept of time. The fact is that all these words now are prevalently used in such meanings. In addition, the semantic extension and interactive use of these time interval words produce new meanings for more specific time expressions. We have a wealth of evidence from ancient Chinese cultural artifacts as well as documentary evidence from Chinese character dictionaries, ancient books and poems to support this. Due to space limitations, what was mentioned above can only cover part of the evidence. In addition, as we mentioned earlier, many of the ancient Chinese methods of reckoning and estimating time, as demonstrated by these time interval words, have been preserved to this day.

## Conclusion

Starting from the wide recognition of space–time mapping in a wide range of languages, with English in particular, sound analysis of solid evidence, more specifically time interval words, confirms our view of event-based metonymic conceptualization of time. The abundant evidence ranges from the cultural origins of the Chinese language to the original meaning and the constructions of time interval words in modern Mandarin Chinese. Relevant cultural and artifactual evidence suggests that event-based concepts of time are pervasive in ancient Chinese culture. It is believed that events and event structure are the fundamental building blocks of the conceptualizations of Chinese ancestors, whose essence was to achieve conceptualizations of cognitive objects in terms of event metonymies. The Chinese words for positioning and measuring time also reflect this cognitive approach, in which temporal landmarks and intervals based overwhelmingly on events. The convincing evidence presented in this paper have also shown that event-based time intervals are organized as complex systems of lexicalized time intervals, anchored and indexed by the annual, seasonal and diurnal rhythmic events in the natural environment, and the regulative rules governing social life and customs, which may be represented by highlighting different aspects of the process of metonymic conceptualization. This evidence suggests that Chinese time interval words are highly persuasive as the linguistic representations of time conceptualization in Mandarin Chinese due to their unique cognitive and cultural functions. The event-based metonymic conceptualization of time as shown in the time interval words strikes the key point of the dual nature of time as time ontology and time attribute or categorial features. The view claimed and consolidated in the paper both reflects the characteristics of Chinese thinking and the historical imprints of the Chinese way of understanding the world, and further justifies the view that Chinese is a metonymic language (Huang, 1995, p. 250).

Further research with larger samples is needed to further confirm these findings. The results of our study shift the previous work on representation of time in Mandarin Chinese in terms of spatial metaphor to event metonymy. In agreement with Galton, we conclude that the spatialization of time through metaphor can never do justice to the fundamental nature of time as a feature of our experience (2011, p. 695). We believe that it is an effective method to explore the time proposition from different cognitive perspectives and explore the reality of the concept of time from the perspective of language and culture.

The event-based metonymic time conceptualization view can find promising application in a variety of research areas like comparative linguistic studies, literature (especially the affinity between Chinese poetry and painting), cultural anthropology, etc. More demanding research should also be done as to when and how metaphorical and metonymic mapping in thinking divides their labor and interact in actual daily communication and thought. Besides, a metaphor–metonymy interaction in the conceptualization of time across cultures is also of great significance.

## Data availability statement

The raw data supporting the conclusions of this article will be made available by the authors, without undue reservation.

## Author contributions

LZ and ZL were responsible for the conceptualization of the study and have approved it for publication. LZ contributed to the data analysis and interpretation. LZ was mainly responsible for the writing of the text with contributions from ZL. Both authors contributed to the article and approved the submitted version.

## Funding

This project was supported by the key research project of the National Social Science research Fund of China (Grant Number: 19AYY012, project ‘A comparative study of syntactic and semantic relations between Chinese and English under temporal and spatial cognitive differences’) and the joint project of Foreign Language Research of Hunan Philosophy and Social Science Fund (Grant Number: [2022] No.5, project ‘Research on the relationship between the conceptualizations of time-space and the subjectivity in Mandarin Chinese’). We would like to thank these funding bodies for their support.

## Conflict of interest

The authors declare that the research was conducted in the absence of any commercial or financial relationships that could be construed as a potential conflict of interest.

## Publisher’s note

All claims expressed in this article are solely those of the authors and do not necessarily represent those of their affiliated organizations, or those of the publisher, the editors and the reviewers. Any product that may be evaluated in this article, or claim that may be made by its manufacturer, is not guaranteed or endorsed by the publisher.

## Footnotes

^1^Shuowen Jiezi (Explanation on Chinese Characters) is the first Chinese dictionary analyzing the shape of the characters and searching the source of characters systematically in ancient China, compiled by Xu Shen in the Eastern Han Dynasty (25-220) and completed in the first year (121) during the reign of Jianguang. The book traced the source of 9,353 Chinese characters recorded one by one, laying the foundation for the lexical works in ancient China. By summarizing the former theory of "Liu Shu" (Six Ways of Character Formation), Xu Shen (1963)created the method to systematically explain characters, i.e. explaining the meaning first, analyzing the structure then, and marking the pronunciation at last. The way to analyze grapheme was unprecedented. Shuowen Jiezi made great contribution to the study on ancient characters, ancient literature and ancient history

^3^ https://baike.baidu.com
